# Learning to Use Narrative Function Words for the Organization and Communication of Experience

**DOI:** 10.3389/fpsyg.2021.591703

**Published:** 2021-03-03

**Authors:** Gregoire Pointeau, Solène Mirliaz, Anne-Laure Mealier, Peter Ford Dominey

**Affiliations:** ^1^INSERM UMR 1093-CAPS, Université Bourgogne Franche-Comté, UFR des Sciences du Sport, Dijon, France; ^2^Robot Cognition Laboratory, Marey Institute, Dijon, France; ^3^École Normale Supérieure de Rennes, Bruz, France

**Keywords:** narrative, situation model, discourse marker, reservoir computing, narrative practice

## Abstract

How do people learn to talk about the causal and temporal relations between events, and the motivation behind why people do what they do? The narrative practice hypothesis of Hutto and Gallagher holds that children are exposed to narratives that provide training for understanding and expressing reasons for why people behave as they do. In this context, we have recently developed a model of narrative processing where a structured model of the developing situation (the situation model) is built up from experienced events, and enriched by sentences in a narrative that describe event meanings. The main interest is to develop a proof of concept for how narrative can be used to structure, organize and describe experience. Narrative sentences describe events, and they also define temporal and causal relations between events. These relations are specified by a class of narrative function words, including “because, before, after, first, finally.” The current research develops a proof of concept that by observing how people describe social events, a developmental robotic system can begin to acquire early knowledge of how to explain the reasons for events. We collect data from naïve subjects who use narrative function words to describe simple scenes of human-robot interaction, and then employ algorithms for extracting the statistical structure of how narrative function words link events in the situation model. By using these statistical regularities, the robot can thus learn from human experience about how to properly employ in question-answering dialogues with the human, and in generating canonical narratives for new experiences. The behavior of the system is demonstrated over several behavioral interactions, and associated narrative interaction sessions, while a more formal extended evaluation and user study will be the subject of future research. Clearly this is far removed from the power of the full blown narrative practice capability, but it provides a first step in the development of an experimental infrastructure for the study of socially situated narrative practice in human-robot interaction.

## Introduction

Meaning is grounded in social and cultural conventions expressed in the forms of words (Waxman and Markow, 1995), grammatical constructions (Goldberg, 2003; Tomasello, 2003), and narrative patterns (Bruner, 1991; Hutto, 2007) that are elaborated through shared experience. Theories of narrative practice hold that through normal exposure to narratives about human social interaction, the child will come to learn how to interpret, react to and respond to social contexts as provided by a theory of mind or folk psychology (Hutto, 2007; Gallagher and Hutto, 2008; Nelson, 2009). This narrative practice theory holds that in human interaction, people regularly generate folk psychological narratives that explain why a person acted on a particular occasion, and that through exposure to these narratives children acquire the skills to understand and themselves produce such narratives (Hutto, 2007). This provides an answer to questions of socially situated language learning - To what extent do representations gleaned from the social and cultural context influence language processing and learning? What mechanisms contribute to socially-situated language processing and learning? The current research provides a theory of how exposure to situations and language describing those situations can be used to establish norms about how language should be used to describe and answer questions about these situations. One method to demonstrate the feasibility of such a theory is in the context of social interaction between humans and robots. The objective of the research described in this paper is to spell out a framework for implementing the theory, and to establish its feasibility in a proof of concept demonstration, leaving more formal and extended user studies for the future.

**FIGURE 1:**
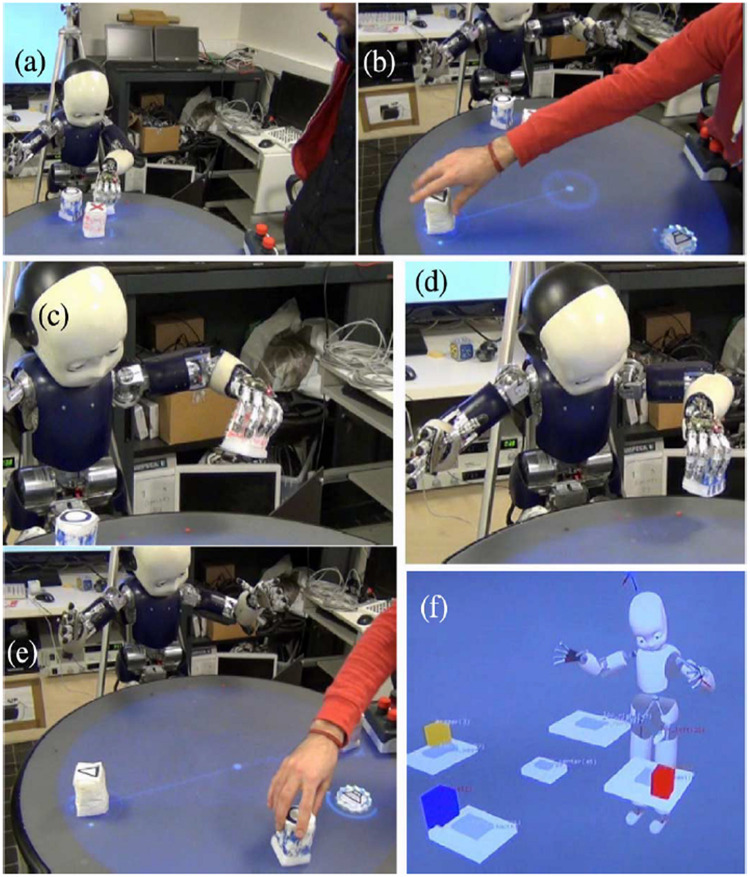
Human-robot social interaction -joint execution of a shared plan that is learned from experience and coded in the autobiographical memory (ABM). Different steps of the iCub during the execution of a shared plan for the music game are illustrated. **(a)** Initial configuration of 3 elements. Robot places object 1 north. **(b)** Human takes this object and places it west. **(c)** Robot places object 2 North, for the human, who then puts it East (not shown). **(d)** Robot places final object north. **(e)** Human takes object and places it South. **(f)** Final internal representation of objects on ReacTable to produce the song as the joint goal of the shared plan. From Pointeau et al. (2014a).

Spoken language has historically played an important role in interactive robot-human communication (Crangle and Suppes, 1994; Lauria et al., 2002; Dominey et al., 2009; Kollar et al., 2010; Matuszek et al., 2013). In the most direct usage, language allows the robot to describe events that have just occurred (Dominey and Boucher, 2005), and allows the human to command actions that the robot should perform (Dominey et al., 2007a,b, 2009). Extending the usage of language in time, we have used spoken language to allow the human to explain a coordinated, cooperative shared plan to the robot, and then to help explain and show the robot how to perform the different actions in the shared plan (Petit et al., 2013; Sorce et al., 2015) as illustrated in Figure 1.

Ideally, however, language allows a much more extended access to events and relations between events and the mental states of the agents involved, as those events occur in extended time. This more extended use of language brings us to something approaching narrative. In her characterization how the child begins to go beyond purely canonical representations of its life events, Nelson states that “Narrative is the vehicle of communicating representations of events between people by verbal means.” [(Nelson, 2003), p. 32]. Nelson specifies that narrative processing requires a grammatical processing capability sufficient to handle the complexity of the sentences used in the narrative, a form of working memory that allows the construction of a representation of the unfolding story, and appropriate experiential memory for encoding and interpreting the situations that the story refers to Nelson (2009).

**FIGURE 2:**
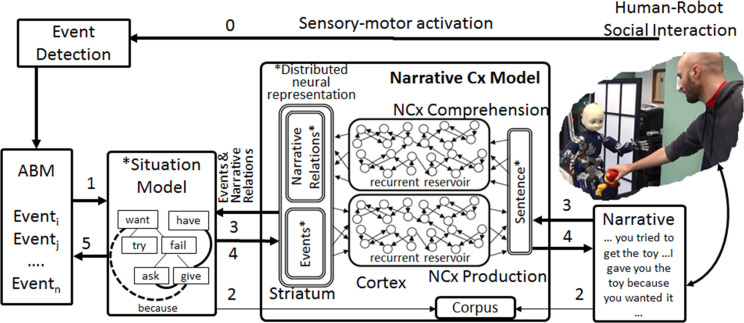
Narrative processing model. Original reservoir computing models for comprehension and production updated with narrative relations in the meaning component to yield Narrative Construction (Cx) Model. Human-robot social interaction generates events (0)_:_ coded in the autobiographical memory (ABM), and (1) transcribed into the situation model. Narrative input maps and meaning representations form a (sentence, meaning) corpus (2) that is used to train the comprehension and production models. Once trained, narrative input is processed by the comprehension model which allows enrichment of the SM via narrative relations (like “because”) that are coded by narrative function words. The system can take contents of the situation model extract the events and narrative relations and use these to generate narrative output (4).

That is, language is about something, and this something is the shared experience of the participants. In this context, we have made a significant effort to develop an autobiographical memory (ABM) system that allows the iCub humanoid robot to store its experience with humans, and to organize this experience in pertinent manner, thus allowing the iCub to learn and perform shared plans for joint action (Petit et al., 2013; Pointeau et al., 2014; Moulin-Frier et al., 2017), as illustrated in Figure 1. This ABM system thus contributes in part to Nelson’s requirement for experiential memory. The requirement for grammar processing can be met with our work in dynamic construction grammar (DCG) (Hinaut and Dominey, 2013; Hinaut et al., 2014, 2015; Dominey et al., 2017). These ABM and DCG capabilities have been integrated in a model of narrative processing (Mealier et al., 2017), illustrated in Figure 2.

Further responding to Nelson’s requirements, we have developed a system where a situation model (Zwaan and Radvansky, 1998; Zwaan and Madden, 2004) is assembled from events coded in the ABM, and is then enriched by linking event representations with causal and temporal relations that are coded by narrative function words. This extends our work on dynamic construction grammar (DCG) using recurrent reservoir networks for sentence processing (Hinaut and Dominey, 2013; Hinaut et al., 2014, 2015). These models are called Dynamic Construction Grammar because of the internal dynamics of the recurrent reservoir network that produces on-line dynamic responses to model inputs, as required for simulating ERP responses (Hinaut and Dominey, 2013). These reservoir computing models learn the relation between the structure of sentences, and meaning, as the mapping of semantic words in the sentences (nouns and verbs) onto their semantic roles of predicate, agent, object and recipient (PAOR). This corresponds to the elements in the Narrative Cx Model in Figure 2.

This model of narrative processing will form the core infrastructure for our study of narrative practice. In the following we outline the extension from grammar to narrative, the elaboration of the situation model, and the use of narrative function words to express relations between event components within the situation model. Then we demonstrate the proof of concept for the learning of how to use narrative function words in responding to questions and in the generation of canonical narrative patterns.

### From Grammatical Construction to Narrative Construction

The extension from the original grammatical construction models is based on the introduction of narrative function words in the sentences, and corresponding narrative relations in the meaning. Whereas grammatical function words (e.g., *to*, *by*, *was*) specify relations between open class words and their semantic roles within a sentence – e.g., who did what to whom - narrative function words specify relations between events in multiple sentences, and their constituent elements at the level of the situation model – e.g., why someone did something to someone.

The original DCG models allowed the learning of the mapping between event meaning and sentences. We then introduce the notion of narrative relations into the meaning. So the sentence “I gave you the toy because you wanted it” corresponds to the meaning with two events gave (I, you, toy) and want (you, toy), linked by the causal relation *because*. This new component of the meaning is labeled “Narrative Relations” in Figure 2. Thus, the recurrent neural network and readout learns to extract the *predicate* (*agent, object, recipient*) (PAOR) representations of events, and the narrative relations. This is the content that can now be constructed into a coherent representation of the narrative, the situation model, based on the narrative construction.

The narrative construction is compositional, built up from multiple sentences that are linked by relations along these dimensions. The nature of such relations and their representation has been identified in various discourse models, such as Centering Theory (Grosz and Sidner, 1986; Grosz et al., 1995), rhetorical structure theory (Mann and Thompson, 1988), SDRT (Lascarides and Asher, 1993), or coherence and structure of discourse (Hobbs, 1985). Taking the analogy from grammatical constructions, these relations are coded by the order of the sentences and by narrative function words (e.g., but, since, then, so, now, because, etc.). The crucial notion is that narrative structure provides a higher level of organization upon the events that it describes. New links—causal, intentional, temporal, etc., and aspects of meaning about people and events that may breach the canonical structure—are superimposed on the events by the narrative discourse, and this structuring results in the creation of meaning referred to by Bruner (1990, 1991, 2009). It is likely that there is a constructive interaction between pre-linguistic representations of such links, and language that labels and highlights these links as the child becomes increasingly proficient (Bruner, 2009).

We have developed methods for representing and expressing meaning about physical events in grammatical constructions (Dominey and Boucher, 2005; Hinaut et al., 2014). The constructions are learned in a manner similar to how humans communicate such meaning in sentences. Paired <sentence, meaning> corpora are created, and used to train the comprehension and production models. This form-meaning learning can be extended to narrative constructions, which allow humans to communicate meaning about a group of events that occurred in a coherent behavioral context, and importantly to express relations between events that may not be visible. Where the grammatical construction uses word order and grammatical functions words to map open class elements onto their thematic roles, the narrative construction uses sentence order and narrative function words to map multiple sentences onto events and relations between them. The form pole of the narrative construction is thus composed of a sequence of sentences that are linked via narrative function words—much like the grammatical function words (closed class words) that provide the grammatical structure at the sentence level (Mealier et al., 2017). Narrative function words have been characterized as discourse connectives which provide discourse structure (Grosz and Sidner, 1986; Knott, 1996; Knott and Sanders, 1998; Fraser, 1999; Webber et al., 2001), much like grammatical function words (closed class words) provide grammatical structure at the sentence level. Norrick (2001) shows how discourse markers “well” and “but” can take on special narrative functions distinct from their lexical meanings and usual discourse marker functions, supporting the psychological validity of the notion of narrative function word.

Narrative constructions are thus learned as conventions (Hutto, 2007), in the same way that grammatical constructions are learned as conventions. As with the grammatical construction model, the system must be furnished with matched sentence-meaning pairs. The novelty is that these sentences will include narrative function words, whose role will also be reflected in the meaning representation. That is, they will be intrinsically present in the sequential structure of sentences and in the meaning representations in training corpora, and learned by the system. Crucially, however, as mentioned above, there may be components of the narrative structure that are not visible in the physical events, e.g., causal and logical relations. These relations will be introduced by the narrator in the narrative examples. This is part of how narrative is used to make meaning (Bruner, 1990, 1991), including the construction of the situation model.

### The Situation Model

**FIGURE 3:**
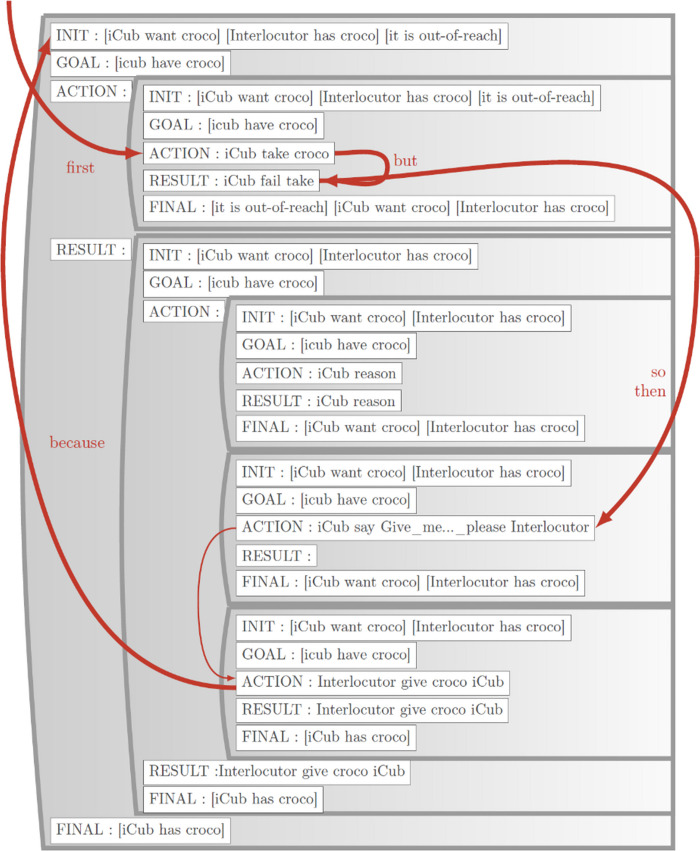
Elaborated Situation model corresponding to the narrative: “*I* wanted to take the croco but I failed the take. So I said give me the croco please. You gave me the croco because I wanted it. Now I have the croco.” The SM is organized in an IGARF structure (Initial state, Goal, Action, Result Final state). The tree-like structure of the SM in Figure 2 is represented here by indentation. This SM was created automatically by the model in Figure 2. From Mealier et al. (2017).

A narrative construction maps multiple sentences onto a situation model, specified as a network of these PAORs (predicate, agent, object, recipient frames), linked by relations along the five dimensions of Zwaan and Radvansky (1998): time, space, causation, motivation, and protagonist. Inspired by psycholinguistics (Zwaan and Radvansky, 1998; Zwaan and Madden, 2004), our situation model codes events, organized around an event structure with Initial state, Goal, Action, Result and Final state – IGARF. These events are linked with narrative relations (causal, temporal, intentional) from successive sentences in the narrative. Recalling from above, this involves an extension of the notion of grammatical construction to narrative construction which in turn involves the introduction of the notion of narrative function words. In analogy to the way in which grammatical function words operate on relations between open class words in a sentence, narrative function words operate on relations between events in a situation model (Dominey et al., 2017; Mealier et al., 2017). Narrative function words including “because, since, then, so, before, after” allow the construction of relations between events in order to construct and enrich a situation model representation of meaning. A detailed situation model in IGARF format is illustrated in Figure 3.

The situation model addresses a major issue we had to resolve which concerned how the DCG model could accommodate multiple sentences that are linked by their narrative structure and contribute to the construction of a coherent meaning representation. The solution was to extend the meaning pole of the DCG model. As illustrated in Figure 2, the DCG models have the meaning pole that continues to contain a representation of the events described in the sentence. In addition to coding the predicate-argument representation of the events, the meaning component is supplemented with an optional representation of the narrative context as coded by a narrative function word. This is indicated as narrative Relations in Figure 2. For example, in the sentence “I gave you the toy because you wanted it,” the meaning component is the standard predicate-agent-object-recipient (PAOR) of the two events gave (I, you, toy); want (you, toy), and the narrative relations component indicates the narrative function word that is now linked to these events. This link is then added to the situation model, as illustrated by the dotted line in Figure 2, and in the more detailed SM representation in Figure 3.

An important aspect of the situation model is that it provides a form of convergence zone between non-linguistic event representations - which can be internal representations of one’s own actions – and linguistic representations of those same actions. Figure 3 provides details of a situation model where the initial content was generated from a human-robot interaction, and the SM was then completed by human narration.

### Integrated Function

In usage, the human and robot [here the iCub (Metta et al., 2008)] interact based on a complication and resolution scenario, where the human helps the robot to achieve its goal. For example, the robot wants a toy. It tries to grasp the toy, and fails. It then reasons on other actions that could be used to achieve the goal, and asks the human for help. The human gives the toy to the robot. These events, as generated by the robot, are coded in its Autobiographical Memory, and automatically converted into the SM representation. This yields an initial SM. The human then narrates what happened, which enriches the representation that has been initiated in the SM. Each sentence in the narrative is matched to the event that it describes. The resulting sentence-meaning pairs are assembled into a corpus that is then used to train the narrative construction (NCx) comprehension and production models. After the comprehension and production models are trained on the resulting corpus, the trained comprehension model can be used to extract the meaning from the narrative. This extracts the events, which are assembled into a situation model (or used to enrich the existing SM), and narrative relations, that are used to create links between events (illustrated as the dotted link “because” between the give and the want actions in Figure 2, and the narrative links illustrated in Figure 3). Narrative relations are identified as those semantic elements that do not have a direct reference in the meaning component (e.g., there is no representation of “because” in any of the events).

Said in a different way, when learning from a narration of an experienced event, events in the sentences are matched with referenced events in the situation model. Those elements that don’t match must then be narrative function words (NFWs). These will used to create links, labeled with the NFW, between events mentioned in the same sentence. For “I gave you the toy because you wanted it,” *gave* and *wanted* in the sentence match with the *gave* and *want* events coded in the SM. *Because* cannot be found in the SM, and so must be considered an NFW. These events and the narrative relation make up the meaning that is paired with the sentence to constitute (with other pairs) the training corpus used to train the reservoir construction models. When this sentence is then presented to the trained model, the model generates the meaning as the events, and the narrative relation, *because*, which is used to create a labeled link between the want and gave events, as illustrated in the SM in Figure 3.

### Learning to Produce Narrative Using Narrative Function Words From Narrative Practice

Given this infrastructure we can see how the SM can be generated from narrative. In order to generate narrative from the SM, we should just go in the opposite direction: the contents of the situation model are used to generate meanings, with the two components – events and narrative relations, and this feeds into the narrative production model. The problem is that for a given situation model there a multiple different forms of sentences that can potentially be generated. Even more difficult, for new situations observed by the robot, the events will be encoded, but not the narrative relations, since they cannot be seen. Like the child, our system has to learn how events in the situation model are linked by causal and temporal relations, which can then be expressed in narrative. This is the problem we address here. This problem is of interest to researchers in developmental psychology, and developmental robotics.

Developmental studies of the acquisition of narrative function words indicate that there is a progression of complexity that typically starts with the use of “and” as an additive marker, then followed by markers for temporal, casual epistemic, object specification, adversative, notice and other complement relations (Bloom et al., 1980). This emergence of discourse connectives is influenced by multiple factors including the conceptual complexity of the relations to be expressed, syntactic complexity of the forms used to express the relations, and the frequency of use in parental input (Evers-Vermeul and Sanders, 2009). Indeed, we should recall the importance of the parental/caregiver influence in the social context of interaction (Dominey and Dodane, 2004).

In this interactive context the child will learn how to express temporal and causal relations in the domain of human motivations for behavior. This problem has been approached by Hutto (2007) and Gallagher and Hutto (2008) in the context of the Narrative Practice Hypothesis. They argue that children engage in story-telling practices with others, and that through this narrative practice they are exposed to –and learn from- examples of how narrative patterns are used to express reasons why people behave as they do. Like the child, our system will learn how to appropriately use narrative function words, based on experience. This experience takes the form of data characterizing how people talk about actions, and what kind of narrative function words they use to establish causal and temporal links between successive actions in a coherent scenario. These data can then be used to teach the system. To respond to this need, we gathered data from naïve human subjects who observe a human-robot interaction, and are then prompted to describe what they have seen. Their use of language then provides data for the learning system. This is described in section II.

Once we have data on how people use narrative function words to link events in narrative, we must render this data usable for the system. For this, we benefit from previous experience with a learning system that collects statistics on how pronouns are used, and generalizes so that the system learns to correctly use pronouns (Pointeau et al., 2014a). Here, we extended this system so that it accumulates statistics on how narrative function words like “because, first, so, then” specifically link different elements in a situation model, in order to talk about action in a meaningful way. This is described in section III.

Once the system has been trained on data from naïve human subjects, the system can then use this knowledge to discuss what happened with the human in a pertinent manner. These results are presented in Sections IV and V.

#### Ethics Statement

Written informed consent was obtained from the [individual(s) AND/OR minor(s)” legal guardian/next of kin] for the publication of any potentially identifiable images or data included in this article.”

## Collecting Data on How People Use Narrative Function Words

Certain dimensions of language structure can be learned through the extraction of statistical structure during exposure to language stimuli (Pelucchi et al., 2009). However, there are dimensions of language learning which require more direct social situation as in learning how to related other’s behavior to unseen goals and motivations. This is where Hutto’s narrative practice is pertinent, as it provides a framework to explain how children learn narrative patterns that explain behavior. Here we set out to initiate a simple modeling of these phenomena of socially situated learning.

**FIGURE 4:**
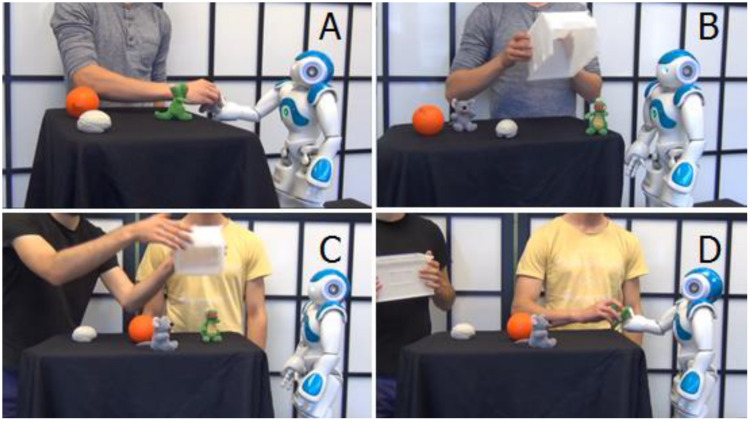
Still images extracted from three of the six videos that were presented to subjects. **(A)** Partner hands an object to the robot. **(B)** Partner removes a box that blocked the robots access to the toy. **(C)** Partner 1 removes the box that obstructs access to the toy, and **(D)** partner 2 then hands the toy to the robot.

In order to determine how people use narrative function words, we invited naïve subjects to watch a series of interactions that involved different levels of “complication” and “resolution,” involving a robot attempting to reach for a toy crocodile and a human helping the robot to achieve the goal. Still images from some of the videos are presented in Figure 4. These scenarios were designed to allow naive subjects to be able to use narrative function words in order to describe when and why the partners and the robot performed as they did. By naïve, we mean that the subjects did not know anything about the algorithms being used to process their responses.

A set of six interactions that involved the human-robot interaction were filmed and put on YouTube, and via Qualtrics we allowed people to access these videos and then describe what they saw. Qualtrics is a tool that allows the creation of experimental protocols that can then be used in web applications such as Amazon Mechanical Turk.

- In Scenario 1, the robot tries to grasp the toy croco, but it is out of reach, so he asks for help, but gets the mouse instead of the croco, and so asks again and this time gets it. - In Scenario 2, the robot tries to grasp the croco that is covered by the box, asks Larry to remove the box, and Robert to give him the croco. - In Scenario 3, the robot tries to grasp the croco, but it is covered by a box, so he asks the human to remove the box, and then grasps the croco. - In scenario 4, the robot successfully grasps the croco. - In Scenario 5, the robot tries to grasp the croco that is out of reach, and then asks for the croco. - In Scenario 6, the robot tries to grasp the croco but it is covered by the box, so he asks Larry to remove the box, then he tries and fails to grasp the croco, and then asks Robert to give him the croco.

In a first data collection we asked people to describe what happened in the videos, using narrative functions words. In these unrestricted cases, the language produced was like this:

(1) The robot seems to fail to pick up the crocodile because he is too far away
(2) So he asks the person because that is easier than moving
(3) The robots takes the time to thank the person, because that is what one should do
(4) The robot finally got the toy
(5) The robot asked the human to give the toy, and finally he could play with it
(6) Although the robot first couldn’t grasp the toy, it finally got it after asking for help
(7) The robot needs help picking the toy because it cannot reach it
(8) It cannot reach it because the toy is far away the user helps the robot because it cannot do it by itself

The NFW system requires that the meaning expressed in the sentences can be associated with meaning in the Situation Model. In some of these example sentences the mapping can be made (e.g., 1, 4, 6), while in others, the sentence refers to meaning components that are not in the SM (e.g., 2, 3). The sentences could be pre-processed, but what we were most interested in was how people used the NFWs to coordinate the main events in the scenarios.

In a second data collection using Qualtrics and Amazon Mechanical Turk, we tested nine subjects in a more structured way where they were given a narrative function word and could select a first event, and a second event, in order to make a sentence that described one of the scenarios. These sentences constructed in this more constrained situation allowed a more direct mapping onto the events in the SM for the discovery of how different NFWs are used to link these events. Here, we used a set of 12 NFW: “and, after, because, before, but, first, finally, however, so, then, therefore, while.” The data collection experiment that was performed by our subjects can be seen on this link:

https://survey.eu.qualtrics.com/jfe5/form/SV_6SF6NuZZdm TCr7D.

Here is an example of naïve subjects use of the narrative function words after, because and before.

The robot ask for the croco to the human after the robot fails to take the croco.

Human gives the croco to the robot because the robot asks the human for the robot.

The robot ask for the croco to the human before the human give the croco to the robot.

**FIGURE 5:**
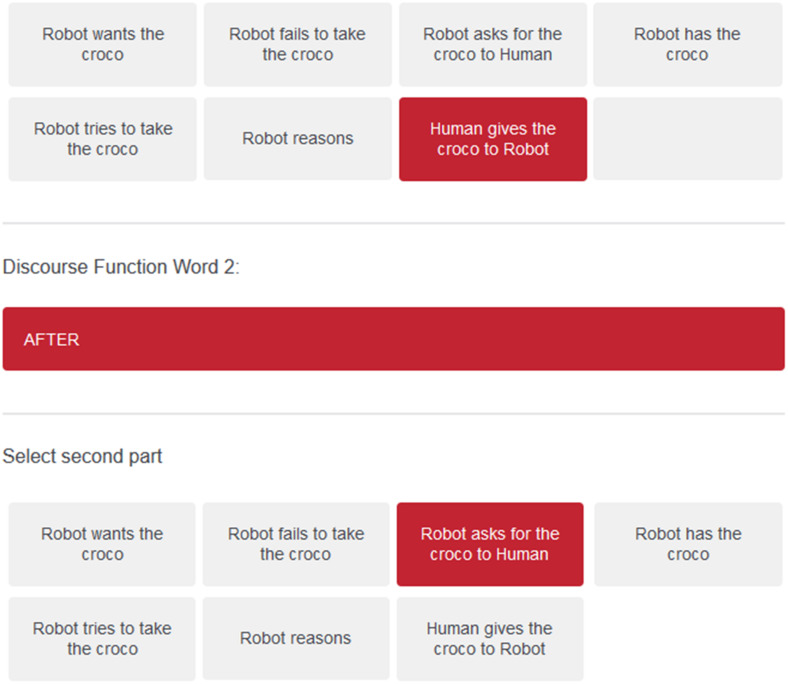
Response panel from user interface for specifying use of NFWs.

A screenshot of the interface for the choice of how to use a NFW is illustrated in Figure 5. This data collection campaign generated 432 distinct uses of the NFWs that could then be used for training the system.

## Learning to Use Narrative Function Words

The situation model represents events and mental (goal) states, and different types of relations between them, expressed with narrative function words. In order to properly generate sentences that express these relations, we took a socially situated usage based approach (Tomasello, 2003) where the knowledge of how to use these narrative function words like “because” comes through narrative practice (Gallagher and Hutto, 2008; Hutto, 2009). We consider this as approximating learning contexts where people provide narrative about what happened, in the same way that caretakers would talk about events with a developing child, who learns by example.

**FIGURE 6:**
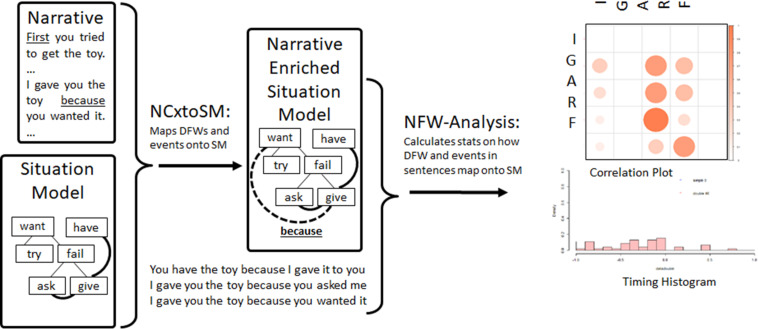
Schematic view of the functioning of NFW-Analysis (Narrative Function Word Analysis). The system calculates statistics on how narrative function words (NFWs) link events in sentence with the corresponding events in the Situation Model of the narrative. Here with the example for the NFW “because”, we see that in the Correlation plot, “because” typically justifies the action of the first mentioned IGARF (Initial state, Goal, Action, Result, Final state - event representation) with any one of the IGARF components of the second event. The timing histogram indicates that the second mentioned event tends to come soon before the first.

Narrative function words express relations between events, and human knowledge about how these relations are expressed is encoded in the data we obtained from human subjects. Through a process of pattern matching statistical learning, the system extracts regularities about how NFWs are used, and then re-employs these statistical patterns when generating narrative. In our algorithm, as illustrated in Figure 6, semantic words in sentences are matched with semantic words representing events in the situation model, in order to identify the referred events. A NFW in a sentence with two events will correspond to a link between these two events in the SM, and this link will be accounted for in the accumulating statistics, represented as the correlation plot in Figure 6. We learn based on two statistical analyses: Correlations between the Initial state, Goal, Action, Result and Final state (IGARF) elements referred to by the NFW, and relative Timing of the elements referred to by the NFW (e.g., does one of the events occur before or after the other).

Table 1 presents the pseudo-code algorithm for extracting this statistical structure. Sentences are analyzed to determine how the narrative function word, and its relation between the event(s) in the sentence, are to be represented in the situation model. For example, we showed that the system can observe that in sentences of the type “event-a because event-b,” there is a relation between the event component (initial state, goal, action, result, final state – IGARF) of the first and second event, and also a relation between the relative timing of event-a and event-b. Statistics on these relations can be accumulated, extending our work in this area for learning how to use pronouns (Pointeau et al., 2014b).

**TABLE 1:** Pseudo-code explaining how narrative function words are learned.

NarrativeFunctionWordLearning(Situation_Model, Narrative)
For each SENTENCE in Narrative
{
MEANING = NarrativeReservoirComprehension(SENTENCE)
// extract meaning with narrative reservoir model
Locate MEANING in Situation_Model
// MEANING may have 1 or 2 events
Establish NFW link with the EVENT(s) in the Situation_Model
//e.g. “because” links Event 1 (Result of IGARF N) with
Event 2 (Action of IGARF M)
Update Correlation_Plot statistics linking IGARF
//e.g. “because” links Event 1 (Result of IGARF N) with
Event 2 (Action of IGARF M)
Update Timing Histogram statistics
// e.g. in this example Event 1 is before Event 2
}

**FIGURE 7:**
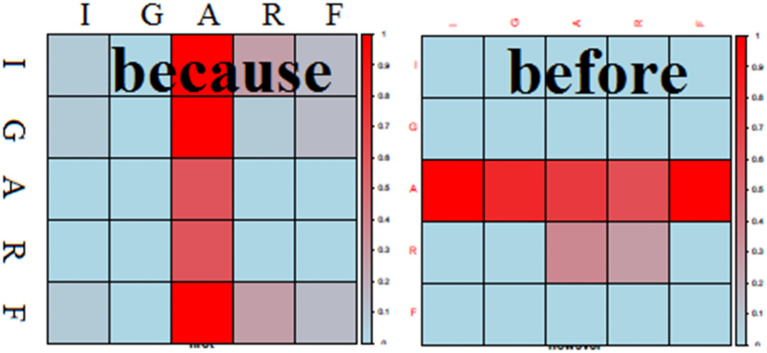
Illustration of correlation plots for IGARF elements of events referred to by the NFW for the first event (horizontal axis) and second event (vertical axis) in data from nine naive subjects. Correlation of the NFW linking the two event elements coded blue (0) to red (100%). Interesting behavior is observed for *because* and *before.* With *because* we see that the action of the IGARF for the first event can be causally linked to all components of a second event, as in the sentence “iCub take the croco because iCub want the croco.” In contrast, the use of “before” can link any element of the first event with the action of the second event.

Figure 7 illustrates the results of this correlation analysis for two NFWs, because and before. We see the typical pattern of usage of these two words, reflected in these statistics. In our data, *because* is most often used to explain why an action occurred, corresponding to the vertical bar along the Action element of event 1. The reason can be related to an initial state, another action, result or final state (e.g., “Anne-Laure removes the box *because* the box cover the mug”). This corresponds to the distribution of probability along the different IGARF elements in the vertical band. For *before*, a different profile is observed. Before is used to explain what happened prior to a particular action (e.g., “the box cover the mug *before* Anne-Laure removes the box.”). What happened before can be any of the IGARF elements, and what happened after is an action. This corresponds to the horizontal band aligned along the Action dimension for before.

## Exploiting Narrative Function Words

The point of learning how NFWs are used is to then be able to use them in this conventional way – to talk about action the way that one has learned that others talk about action. So in answer to a question like “what happened first?” one can respond in the same way that one has heard others talking and using the word “first”. Likewise when asked why did an event occur, one would respond by using the word “because” in the same way that one has seen others do it.

**FIGURE 8:**
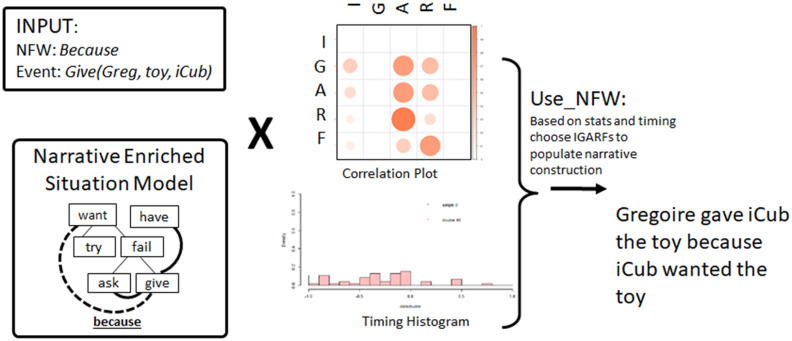
Use_NFW procedure that takes an NFW and an event, and a SM as input, uses the NFW statistics to find an event in the SM that has the NFW link with the input event, and then uses the language model to generate the corresponding sentence.

Use_NFW is a procedure (described in Table 2 and Figure 8) for extracting event representations in the form of IGARFs from the SM based on narrative links encoded in the statistics in the correlation plot and timing histogram learned from experience. The Correlation Plot encodes the source and target events typically referred to by a narrative function word (e.g., “because” often refers to the Action of the source and the multiple possible IGARF elements of the target). The Timing Histogram describes the temporal precedence for source and target.

**TABLE 2:** Pseudo-code explaining how narrative function words are used in responding.

Use Narrative Function Word (pseudo-code)
UseNarrativeFunctionWord (Situation_Model, NFW, input_event(optional))
If input_event <> null
Find input_event in Situation_Model
For each EVENT in Situation_Model
// calculate statistics on most probably use of events
// in SM with this NFW
{
Correlation = score(EVENT(i), input_event, Correlation_Plot(NFW))
Timing = score(EVENT(i), input_event, Timing_Histogram(NFW))
Update score_vector(i)(EVENT, Correlation_score, Timing_score)
}
Response = select_best_event(score_vector)
Sentence = NarrativeGenerationModel(Response)
Say(Sentence)

The function Use_NFW exploits the statistics learned in the correlation plot and the timing histograms in order to determine what elements of the situation model should be extracted and used to generate a meaning representation for the narrative construction production reservoir model. Use_NFW takes as input [NFW, optional event [PAOR], optional order). The optional event is because some NFWs do not take an event (like *first*, or *finally*), whereas others do (like *before* event, or *after* event). The optional order corresponds to the place of the given [PAOR] in the desired sentence: “What happened after I gave you the toy?” (Result: “After you gave me the toy, I have the toy.”) versus “I gave you the toy after what?” (Result: “You gave me the toy after I asked for it”). The function Use_NFW will thus return a set of events with a corresponding score (based on the distribution of the histogram and correlation plot), that will be sent to Narrative Construction Model to be generated as a sentence.

Narrative function words can also be used to search for a particular situation model. That is, a relation expressed by an NFW and one or more IGARFs can be used as a pattern that will be searched for in a set of SMs. This allows a form of interrogation of the system as: “Do you remember when …?”

## Human-Robot Interaction

Once the system has been trained as described above, it is almost ready to use the acquired knowledge in order to communicate about actions. The final element is another form of socially situated knowledge about conventions for how to answer questions. We believe that these conventions (specified in Table 3) can also be learned by narrative practice, but in the current demonstration they are pre-specified as described below.

**TABLE 3:** In order to generate event and NFW inputs for narrative to be generated by the system, we developed a simple mapping between questions that the user can pose, and the corresponding query that will be made to the situation model.

Question	Characteristics of returned events
What happened <first, then, finally>	Return events that respect the statistical (correlation and temporal) characteristics of the identified NFW

What happened <because, after, before> EVENT 1	Return events that respect the statistical (correlation and temporal) characteristics of the identified NFW and the cited event

Why did EVENT	Return events that respect the statistical (correlation and temporal) characteristics of the NFW “because” and the cited event

What else	CONTINUE with the same search

Why is that	Return events with a “because” link to the previously returned event

Do you remember when <first, then, finally> EVENT or EVENT <first, then, finally>	Search for a SM that contains the specified EVENT
	

**FIGURE 9:**
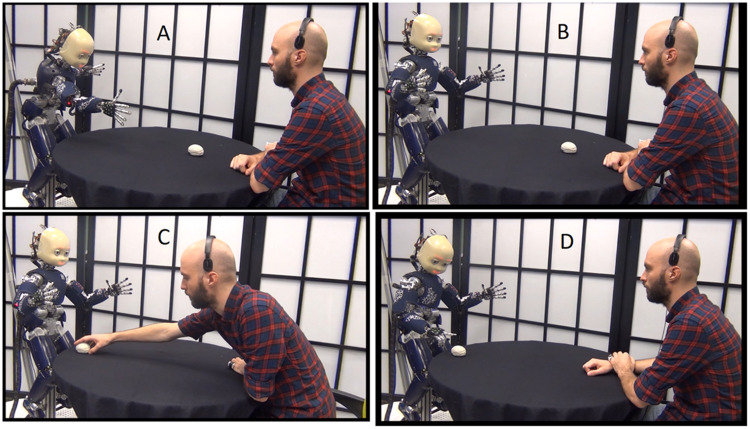
New scenario - trying to take the brain. **(A)** iCub wants the brain and tries to take it. **(B)** After failing, and reasoning, iCub asks Gregoire to give him the brain. **(C)** Gregoire gives the brain to iCub. **(D)** iCub acknowledges that it has the brain.

Here we illustrate the ability of the system to use narrative function words in order to respond to questions in an interactive dialogue with the human. In the examples we present, an interaction first takes place, where the iCub wants to grasp a toy brain (see Figure 9). It attempts the grasp and fails. It then uses reasoning to determine if there is another method to get the brain, and determines that it can ask Greg. It does so, and Greg gives the iCub the toy brain.

The remarkable point here is that there has been no specific training for this scenario. That is, Greg and the iCub experienced a shared cooperative activity where Greg helped the robot, but there was no specific language training about this interaction. Rather, based on several previous interactions, and narrative provided by people, the system was able to learn (as just described) how people use narrative function words like *first*, *then*, *because*, and *finally* – with respect to the ordering and relation between events in a situation model – when talking about events. So, the system learns that when using the word *first* one talks about things that occurred near the beginning of the scenario. When using the word *because* and an event, one talks about events that preceded the event in question.

In order to allow dialogic interaction a simple turn taking setup and a set of questions were developed. The questions and the selection of events that are used to generate the responses with the narrative reservoirs are depicted in Table 3. For an example of the functioning of the system identified in Table 3, consider the sentence “Why did Greg give the brain to you?” This is interpreted as “Why did EVENT” in Table 3. The interpreter thus calls the function Use_NFW to identify events that have a “because” link with the event Give (Greg, brain, iCub) in order to generate a sentence “Greg gave the brain to me because EVENT.” The system will look for events that precede the target event, and that correspond to an Initial state, Action, or Result.

The two dialogues illustrated in Tables 4, 5 were generated in on-line question answering with the system (see video)^1^. We can observe that by the open-ended possibility of questions, the human is allowed to explore the past experience from different approaches. In the example illustrated in Table 4, the human starts at the beginning, by asking “What happened first?” and then moves forward from there. In the second example, Table 5, the human starts at the result by asking “What happened finally?” and works back through the causal chain.

**TABLE 4:** Example dialogue excerpt from an interaction with the iCub.

H: What happened first?
R: **First** I want the brain
H: What else?
R: **First** Greg have the brain
H: What else
R: **First** I take the brain
H: What happened then?
R: **Then** I said: “Give me the brain please” to Greg
H: What else?
R: **Then** Greg give the brain to me
H: Why did Greg give the brain to you?
R: Greg give the brain to me **because** I say: “Give me the brain please” to Greg
H: What happened after Greg gave the brain to you?
R: **After** Greg give the brain to me, I have the brain

**TABLE 5:** This dialogue example illustrates how the human can start at the end of the interaction and ask questions proceeding from end to beginning.

H: What happened finally?
R: **Finally** I have the brain
H: Why do you have the brain?
R: I have the brain **because** Greg give the brain to me
H: Why did Greg give the brain to you?
R: Greg give the brain to me **because** I say ‘give me the brain please” to Greg
H: Why did this happen?
R: I said “give me the brain please” to Greg **because** I failed the take
H: What else?
R: I said “give me the brain please” to Greg **because** I reasoned
H: Why did your reason?
R: I reasoned **because** I failed the take
H: Why did you take the brain?
R: I take the brain **because** I want the brain

In both of these examples, the system did not receive specific training in the scenario that was described in the dialogue. Rather, it was trained on data generated by naïve subjects in different scenarios. This training allowed the extraction of statistical rules about how NFWs are used, which could then be applied to this new situation.

## Generalization of a Canonical Story Template to New Situations

This capacity to extract the rules of use of narrative function words, based on experience encoded in narrative that the robot has heard, allows the system an unprecedented level of generalization that we did not see with the grammatical constructions. In the narrative constructions, narrative function words like “*because*” specify relations between event components within a situation model. The system learns how *because*, and other NFWs, are used. An example is illustrated in Table 6, where a new situation is encountered and used to construct a situation model, then the system applies a learned sequence of NFWs to this situation model to generate a new simple narrative.

**TABLE 6:** Narrative generated from a novel situation, by applying a stored set of NFWs (marked in bold) from a set of questions posed by the human to yield a narrative template.

**First** Anne-Laure have the mouse.
**Then** I said: Give me the croco please, to Anne-Laure.
**Then** Anne-Laure give the mouse to me.
**Finally** I have the croco.
I have the croco **because** I have the mouse.*
I have the croco **because** I said: Give me the croco, to partner

In contrast, in the grammatical constructions, grammatical function words like “by” specify relations between open class elements in the sentence, but rather than learning these relations as statistical rules associated with each grammatical function word (GFW), we learn an entire mapping from the whole sentence, with the global pattern of GFWs, onto the predicate-argument representation of the meaning. Thus, it is impossible for the system to learn the functions of individual GFWs. Interestingly, however, when the DCG model is exposed to sufficiently large corpora, it is able to generalize to new grammatical constructions that it was not trained on Hinaut and Dominey (2013). Thus, functionally, it learns how to interpret grammatical function words in a general manner. Still, the generalization on NFWs in the narrative model is much more powerful, and based on the compositionality of representations in the SM (Dominey, 2003).

However, the generalization is not entirely immune to error. We see with the sentence marked with “^∗^” that the iCub says “I have the croco because I have the mouse.” This is not entirely wrong – prior to getting the croco the iCub did have the mouse. Part of the definition of the *because* relation is that the causal event does precede the caused event, and so from the temporal perspective this error can be understood. Interestingly, this kind of anomalous use of “because” is observed in situations where children (3 year, 6 month–9 year) narrate their personal experience. In the kinds of errors that these children can make, one event follows another, but the first event does not cause or enable the second, or vice versa: e.g., “I fell and just hurted my neck. Because I had to go to the doctor’s to get the shot for my mumps” (McCabe and Peterson, 1985). While it is highly probable that these observations in children reflect cognitive processes we do not model here, still, for children and our system, there is an observation of using because to preserve temporal order where causation is not directly present.

## Discussion

In its ecological form language is highly socially situated and is indeed a vehicle for social situation. Human interactions and events form the social matrix that is observed and must be explained and justified to others. Yet at the same time, the manner in which this communication is to be achieved is itself a social norm that is socially situated in its acquisition. Interestingly, such social conventions apply at the lexical, grammatical and narrative levels, in the service of meaning.

Actions typically do not take place in isolation, and when we talk about actions and events, we don’t simply state dully the action that took place, but instead we talk about the event in an interesting, pertinent way in the ongoing dialogical or narrative context in a way that has *meaning* (Bruner, 1990). We stress the notion of meaning, because meaning is derived not just from the action itself, but from the situated social and narrative context in which it is embedded: why did you do it, who did it, and when! This meaning is characterized by how an action is integrated into an intentional network of interrelated actions. These relations are described by a category of words that we refer to as narrative function words.

Crucially, the manner in which these words are used to express the organization of events and the relations that interconnect them are not arbitrary, nor are they innate. Rather, they are cultural artifacts that are transferred to the young new member of the culture (Tomasello, 1999), through narrative practice (Hutto, 2007; Gallagher and Hutto, 2008; Nelson, 2009; Hutto and Kirchhoff, 2015).

We emphasized how narrative practice could allow children to construct relations between events. A complementary account is that these relations (e.g., causal relations) are already perceived by the infant, and narrative practice allows the infant to learn how to appropriately label and refer to such relations. Our model is consistent with both accounts, and in human development it is likely that there is an interaction between them. Indeed, Bruner suggests that children may initially be limited in this causal paradigmatic thinking, and that adult discourse has a role in guiding children toward the right causal analyses, so that ultimately the child can perform these causal analyses autonomously (Bruner, 2009). Similarly, Lagerwerf (1998) considers that the use of the causal connective “because” presupposes an understanding of the relation between the causing and caused. Further supporting this position (Knott, 1996), considers that coherence relations like causality describe cognitive constructs that we use to represent the world, independent from linguistic processing. Thus, narrative would serve to label and make explicit cognitive constructs like causality that have already been perceived by the child. In Section II we sought to characterize how people use NFWs in the context of the scenarios that we study. This involved data collection in which naïve subjects narrated human-robot interactions, using narrative function words. We found that in fully unconstrained conditions, the sentences generated by subjects did not sufficiently map onto the events that were represented in the situation models, and so we modified our data collection so that subjects were more constrained to generate sentences that refer to events in the situations models and the relations between them, thus providing more concrete demonstrations of how to use the narrative function words.

We then presented algorithms for learning how NFWs are used to express narrative relations about actions in section III, and for using this knowledge to allow the system to then use this knowledge in section IV, with demonstration in section V. At this point we made a remarkable observation about generalization and narrative function words: by learning how specific NFWs are used, the system is able to generalize to new situation models. That is, the learned narrative ability can be applied to contexts different from those used for learning.

In section V we thus demonstrate this ability for the system to communicate about action. The iCub is able to answer questions about diverse scenarios (illustrated here with one), based on the NFW processing.

The system is able to talk about actions in a rather advanced way. Rather than talking about actions in an isolated manner, with each action being independent, instead the system is able to situate actions in time, and with respect to other actions. Likewise, the human can approach the scenario in question from different perspectives – starting at the beginning and working forward, at the end and working backward, etc. This extends our work on perspective and construing an event in different ways (Mealier et al., 2016). This work on learning to use narrative functions words represents an advanced level of human-robot communication about actions. Future work will examine structural relations between situation models, and use of narrative function words in the context of specific (vs. statistical) use in these situation models.

One of the major limitations of the current research is illustrated in Table 3, which provides the form of responses to be used for different types of questions. The limitation is that this type of correspondence is exactly the kind of knowledge that can be acquired through narrative practice. That is, the child or learning system can observe what are the types of responses that are the social conventions for different types of questions. In the current research, this is a limitation in terms of what was actually done, but not in terms of what is theoretically possible.

This raises the point of the final remark in terms of limitations. This research demonstrates a form of feasibility or proof of concept for the ability to learn to use narrative function words in order to organize and communicate experience. As stated at the outset, the behavior of the system is demonstrated as a set of illustrative behavioral interactions, and this proof of concept lays the foundation for a more extensive user study that will be the subject of future research.

## Data Availability Statement

The raw data supporting the conclusions of this article will be made available by the authors, without undue reservation.

## Ethics Statement

Ethical review and approval was not required for the study on human participants in accordance with the local legislation and institutional requirements. The patients/participants provided their written informed consent to participate in this study. Written informed consent was obtained from the individual(s) for the publication of any potentially identifiable images or data included in this article.

## Author Contributions

All authors conceived the system, performed the experiments, and wrote the manuscript. GP, A-LM, and SM developed the software infrastructure.

## Conflict of Interest

The authors declare that the research was conducted in the absence of any commercial or financial relationships that could be construed as a potential conflict of interest.

## Footnotes

1 https://youtu.be/Lhs2aQ7zLK4
